# Supplementary material for: Exosomes Derived from Yak Follicular Fluid Increase 2-Hydroxyestradiol Secretion by Activating Autophagy in Cumulus Cells
Source: Animals (Basel). 2022 Nov 16;12(22):3174. doi: 10.3390/ani12223174 (PMC9686841; doi:10.3390/ani12223174)
Supplement: Supplementary file 1 [file animals-12-03174-s001.zip › Figure S2.pdf]

Yak follicular fluid can activate autophagy in YCCs.

C: Control

E: Yak follicular fluid exosomes

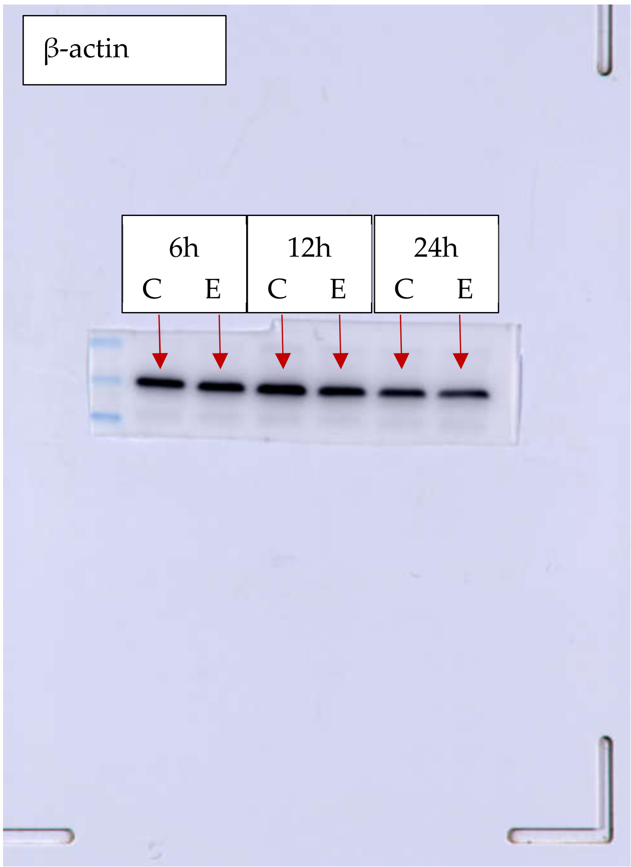

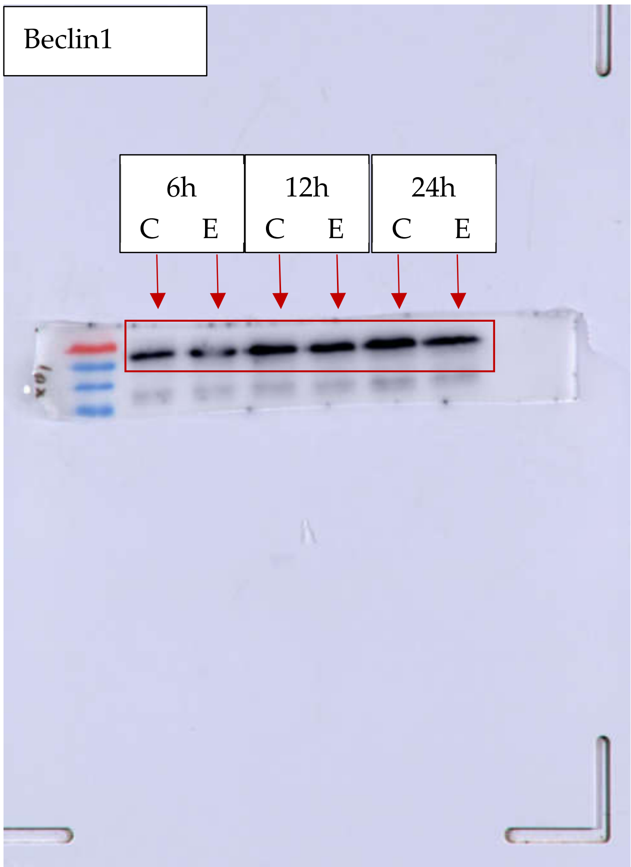
Beclin1
6h
C E
12h
C E
24h
C E

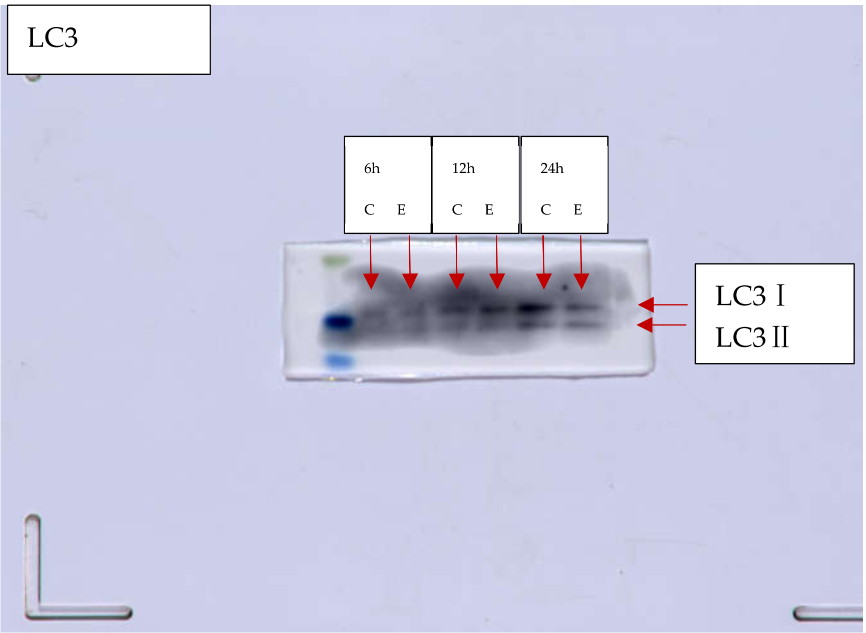
LC3
6h
C E
12h
C E
24h
C E
LC3 I
LC3 II

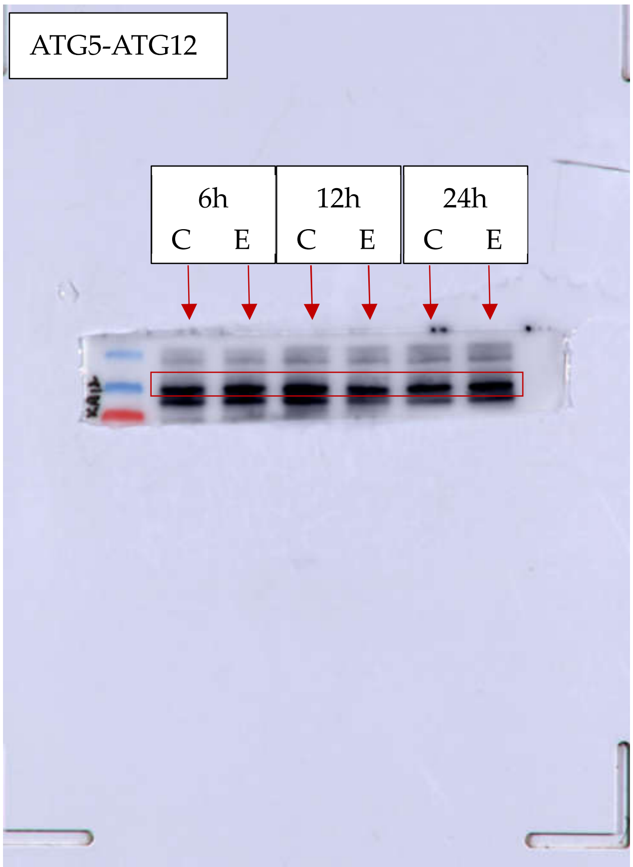
ATG5-ATG12
6h
C E
12h
C E
24h
C E

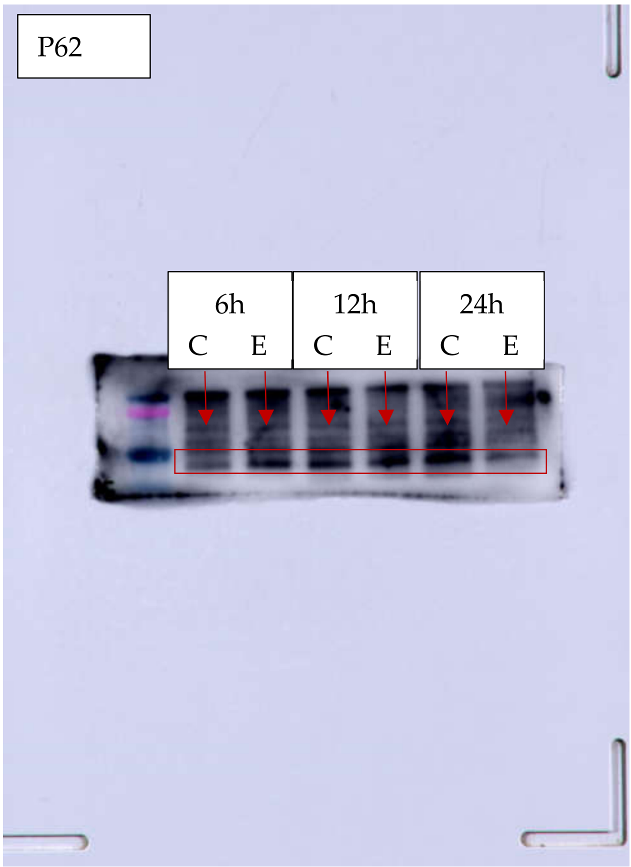
P62
6h
C E
12h
C E
24h
C E

Yak follicular fluid exosomes could increase 2-OHE2 secretion in YCCs.

C: Control

E: Yak follicular fluid exosomes

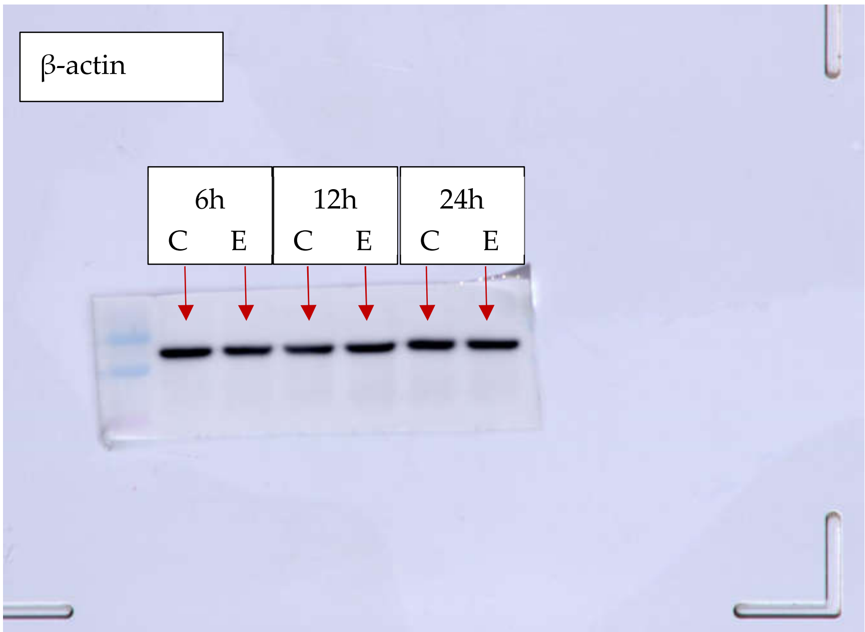

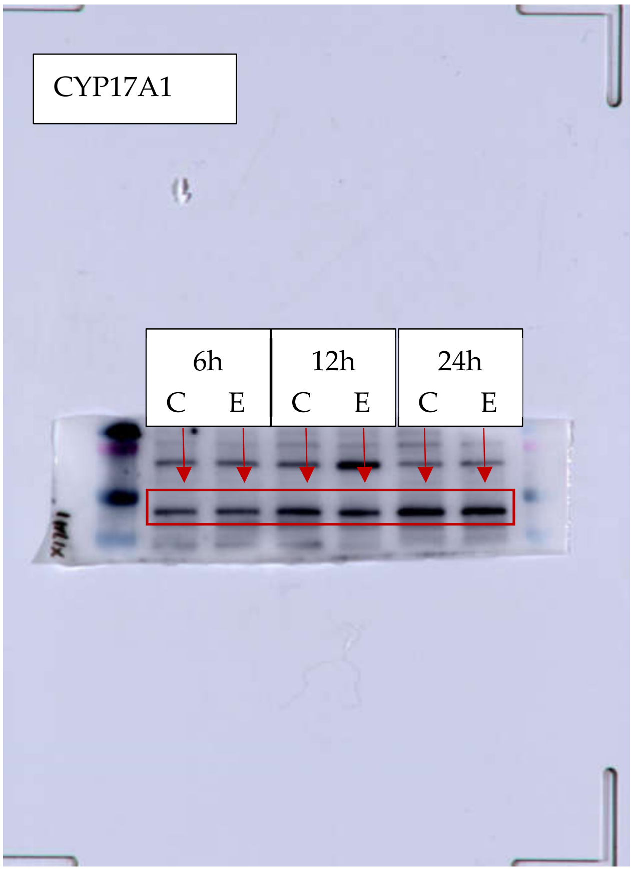

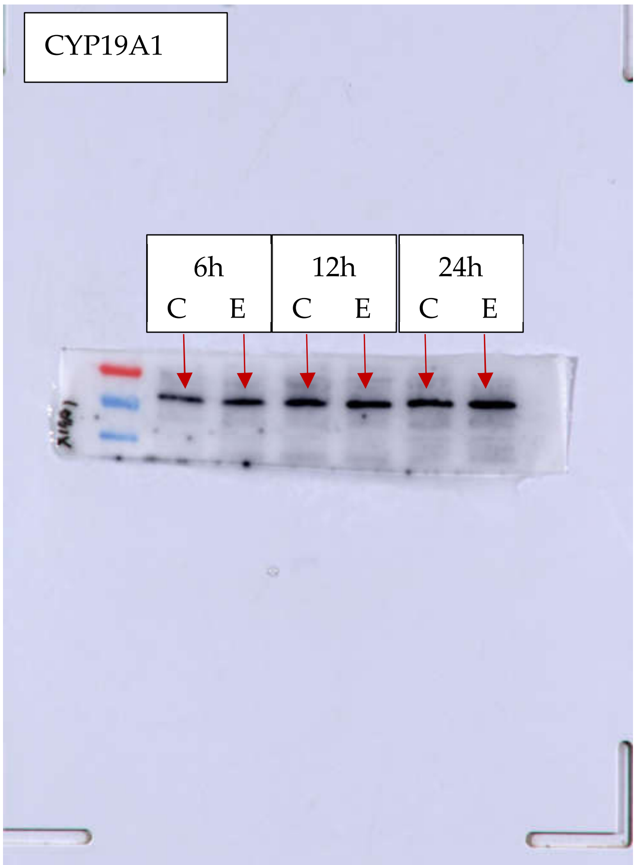
CYP19A1
6h
C E
12h
C E
24h
C E

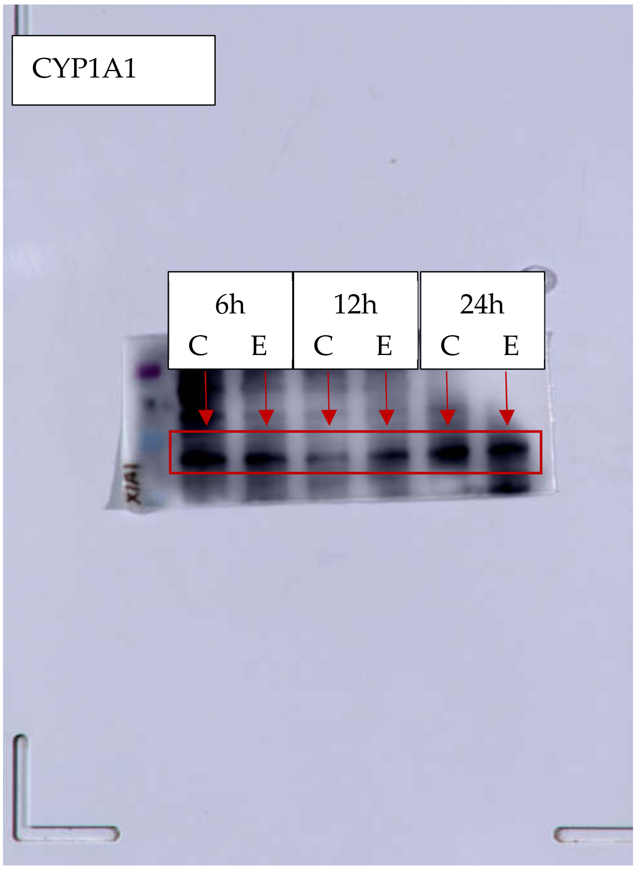
CYP1A1
6h
C E
12h
C E
24h
C E

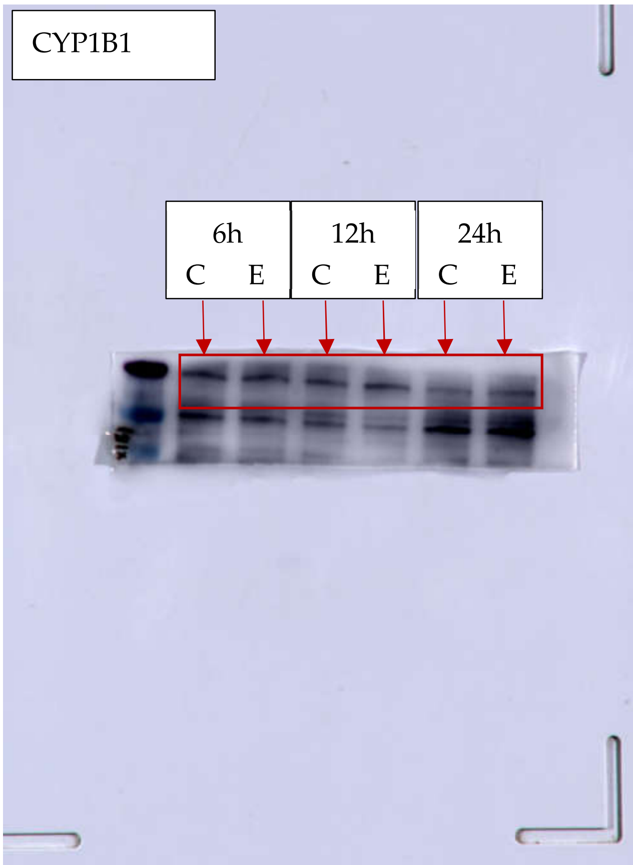
CYP1B1
6h
C E
12h
C E
24h
C E
