# Supplementary material for: Exosomes Derived from Yak Follicular Fluid Increase 2-Hydroxyestradiol Secretion by Activating Autophagy in Cumulus Cells
Source: Animals (Basel). 2022 Nov 16;12(22):3174. doi: 10.3390/ani12223174 (PMC9686841; doi:10.3390/ani12223174)
Supplement: Supplementary file 1 [file animals-12-03174-s001.zip › Figure S3. .pdf]

**3-MA inhibits autophagy and reduces 2-OHE$_2$ secretion in YCCs.**

C: Control

E: Yak follicular fluid exosomes

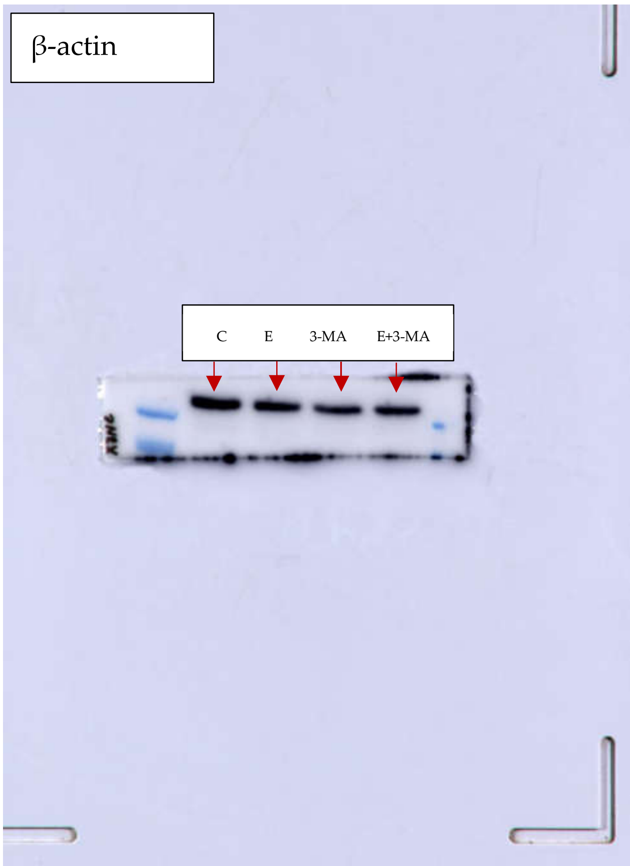

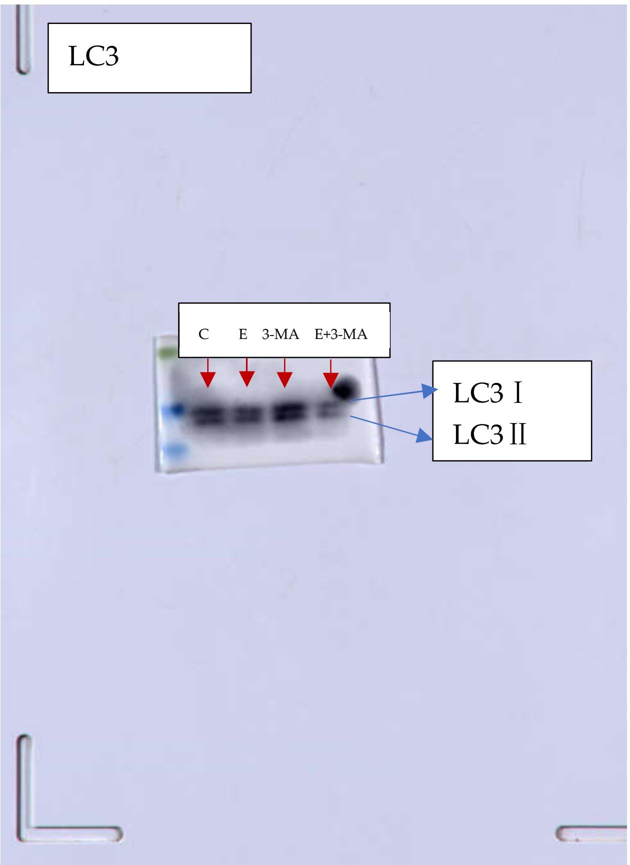
LC3
C
E
3-MA
E+3-MA
LC3 I
LC3 II

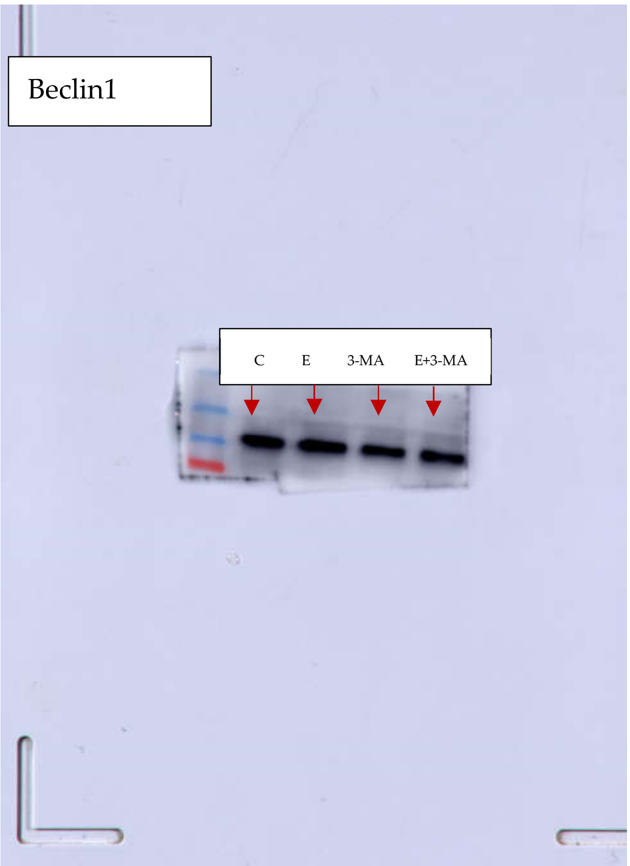
Beclin1
C
E
3-MA
E+3-MA

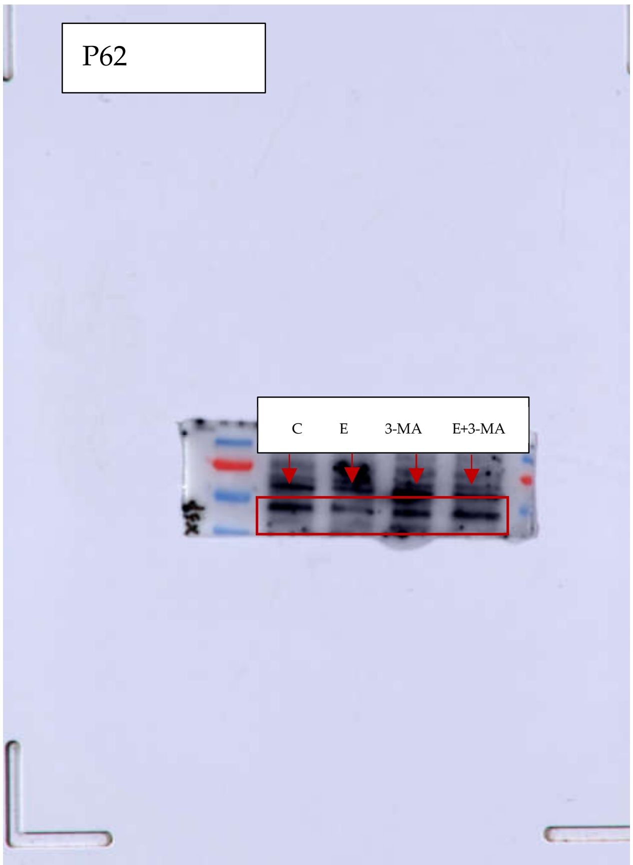
P62
C
E
3-MA
E+3-MA

**2-hydroxyestradiol secretion-related proteins**

C: Control

E: Yak follicular fluid exosomes

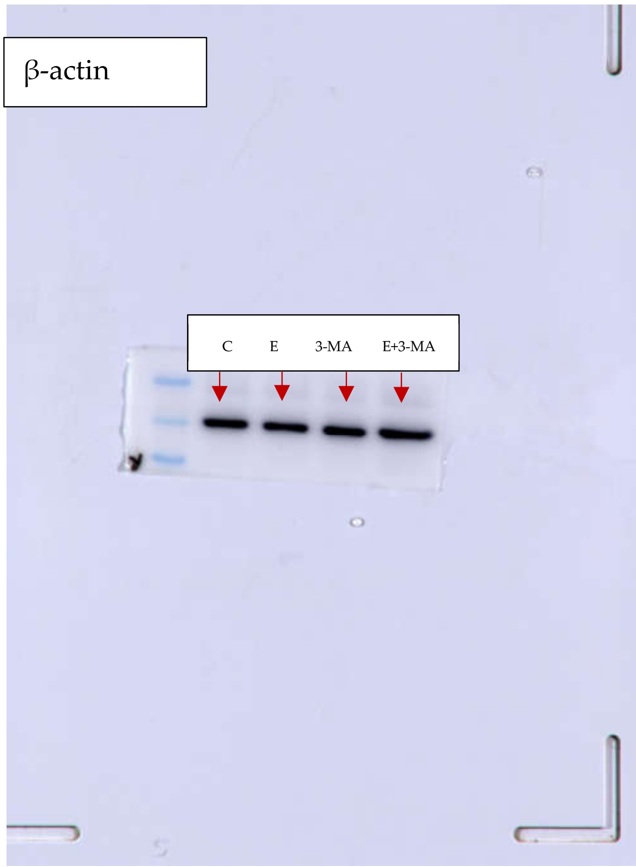

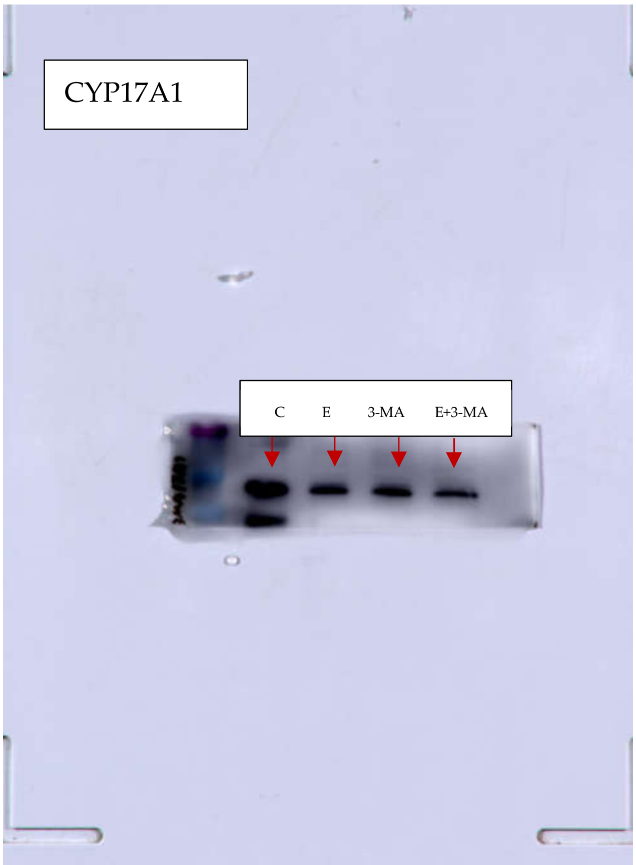
CYP17A1
C
E
3-MA
E+3-MA

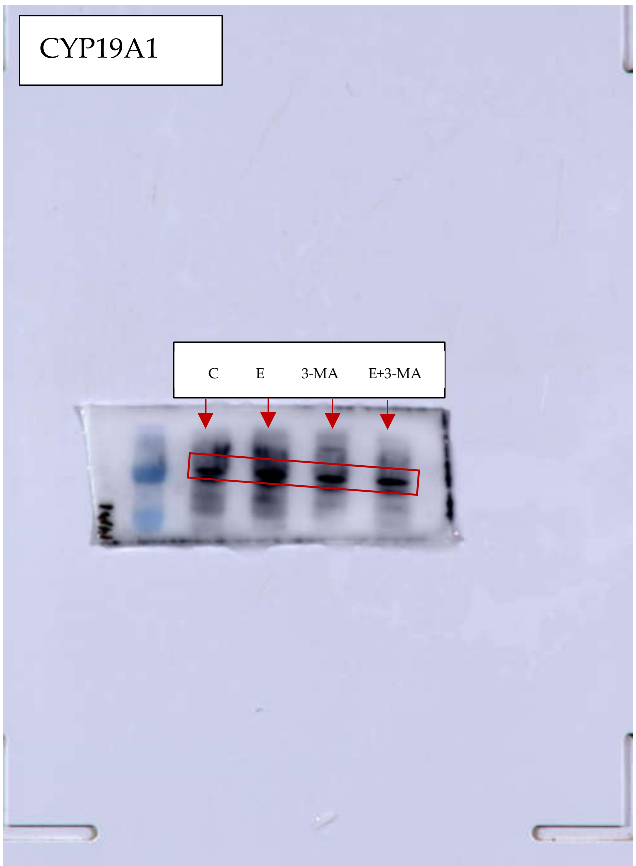
CYP19A1
C
E
3-MA
E+3-MA

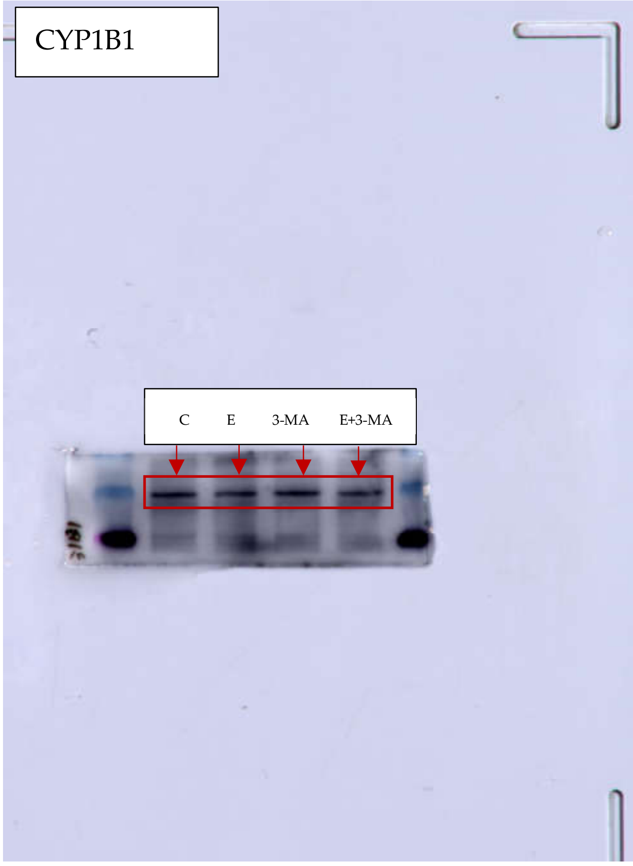
CYP1B1
C
E
3-MA
E+3-MA

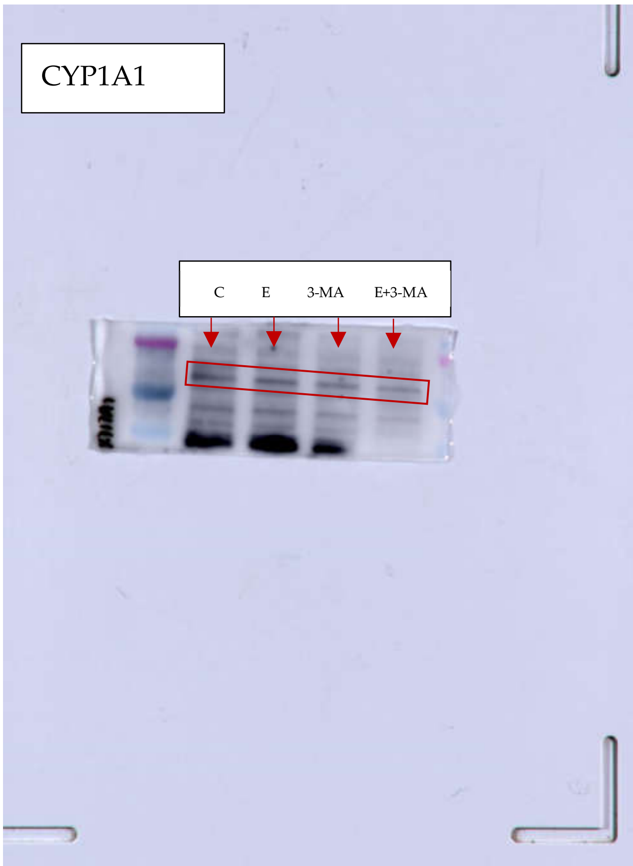
CYP1A1
C
E
3-MA
E+3-MA
