# Supplementary material for: Exosomes Derived from Yak Follicular Fluid Increase 2-Hydroxyestradiol Secretion by Activating Autophagy in Cumulus Cells
Source: Animals (Basel). 2022 Nov 16;12(22):3174. doi: 10.3390/ani12223174 (PMC9686841; doi:10.3390/ani12223174)
Supplement: Supplementary file 1 [file animals-12-03174-s001.zip › Figure S4.pdf]

RAPA increases 2-OHE$_2$ secretion by upregulating autophagy in YCCs

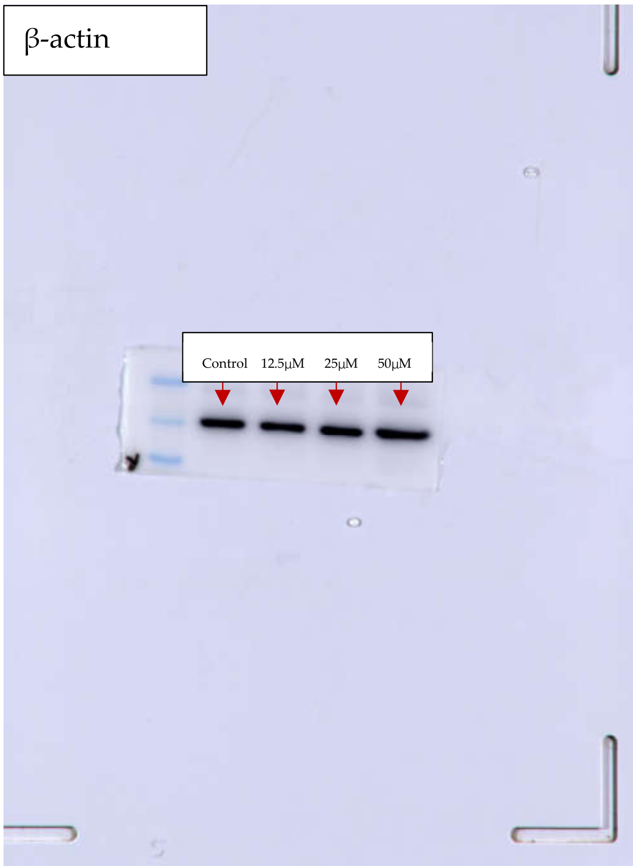

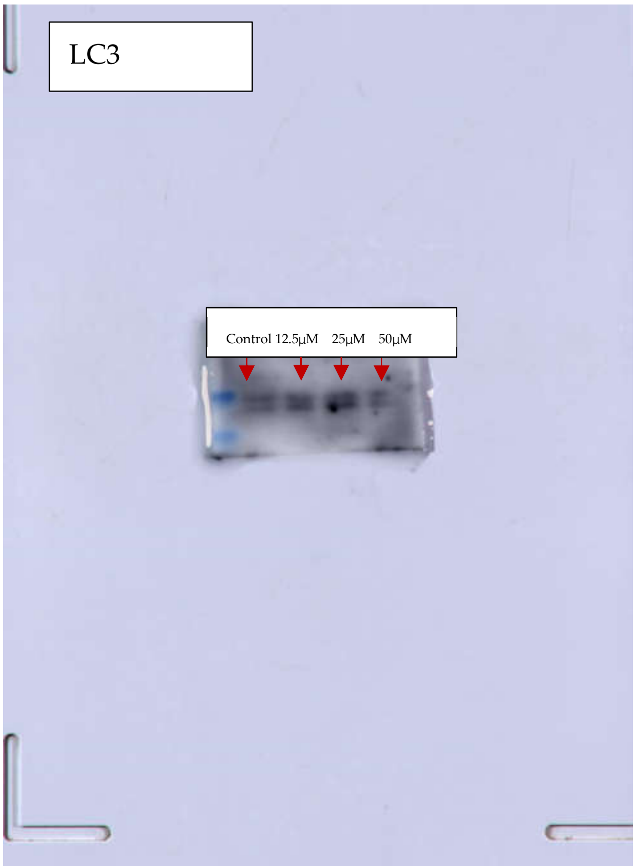

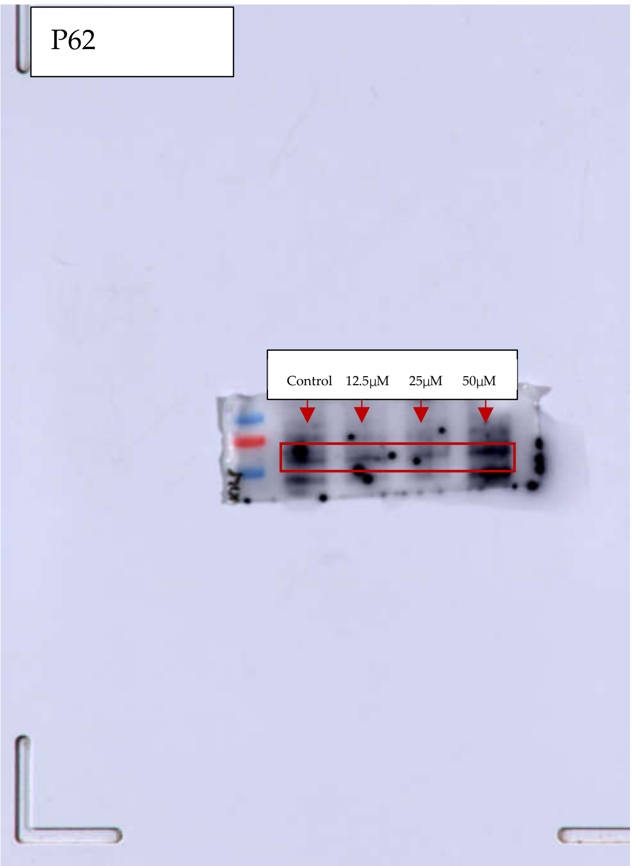
P62
Control 12.5μM 25μM 50μM

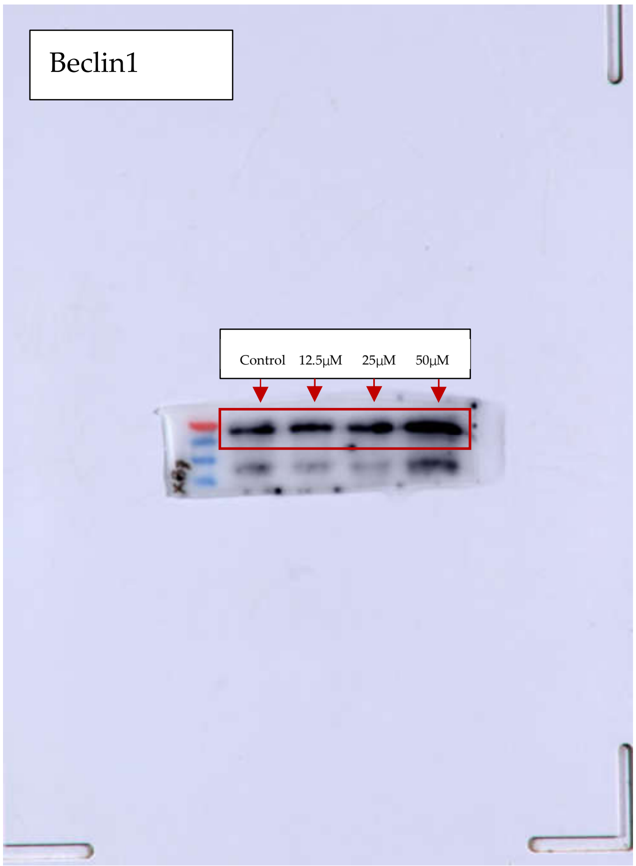
Beclin1
Control 12.5μM 25μM 50μM

2-hydroxyestradiol secretion-related proteins

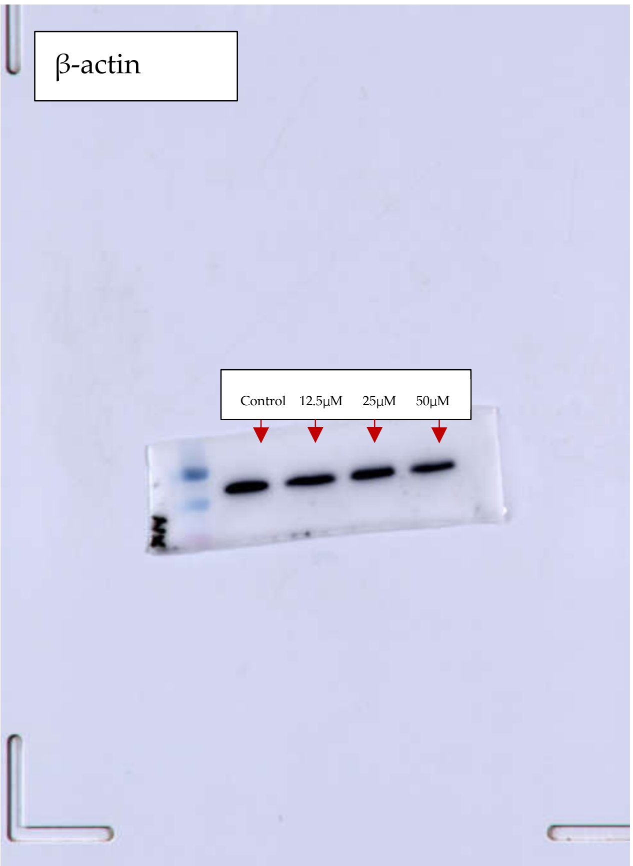

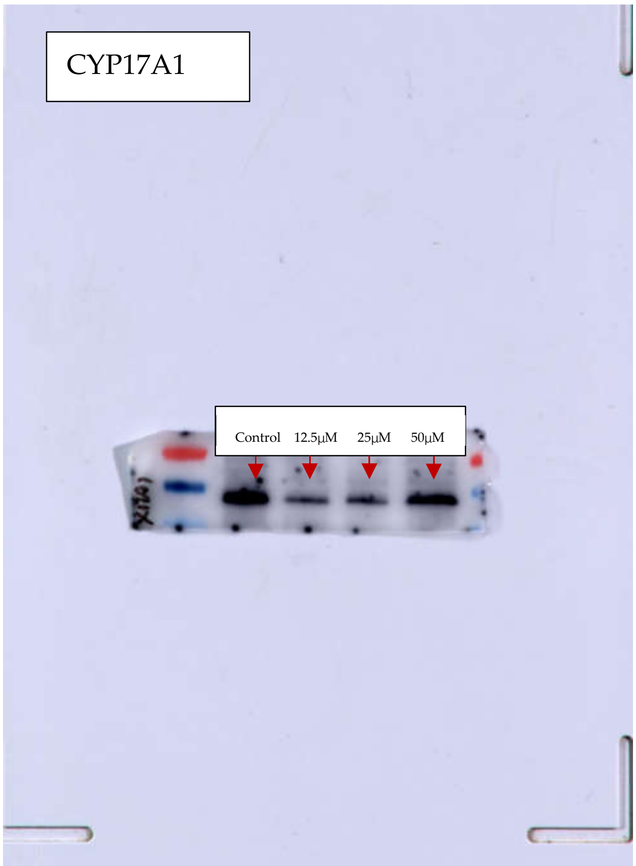

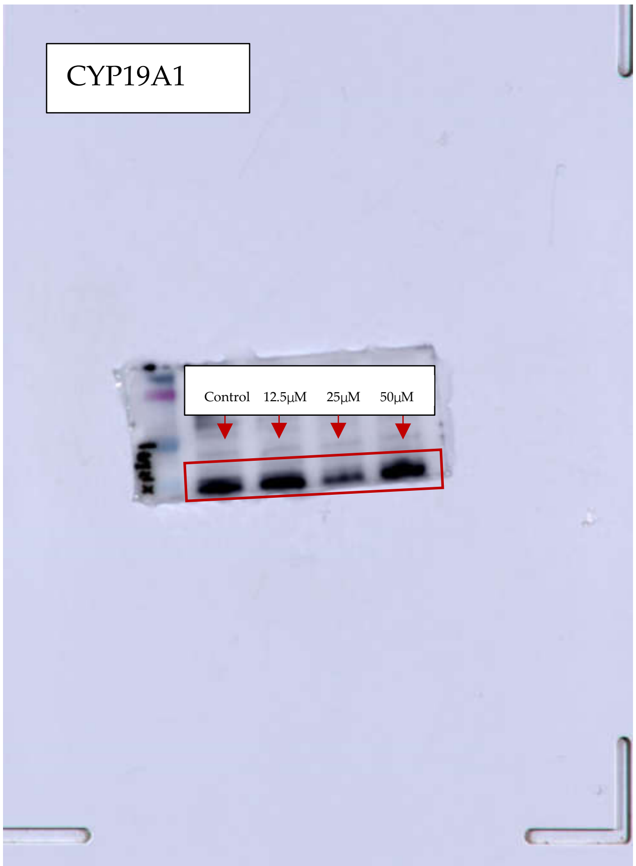
CYP19A1
Control
12.5μM
25μM
50μM

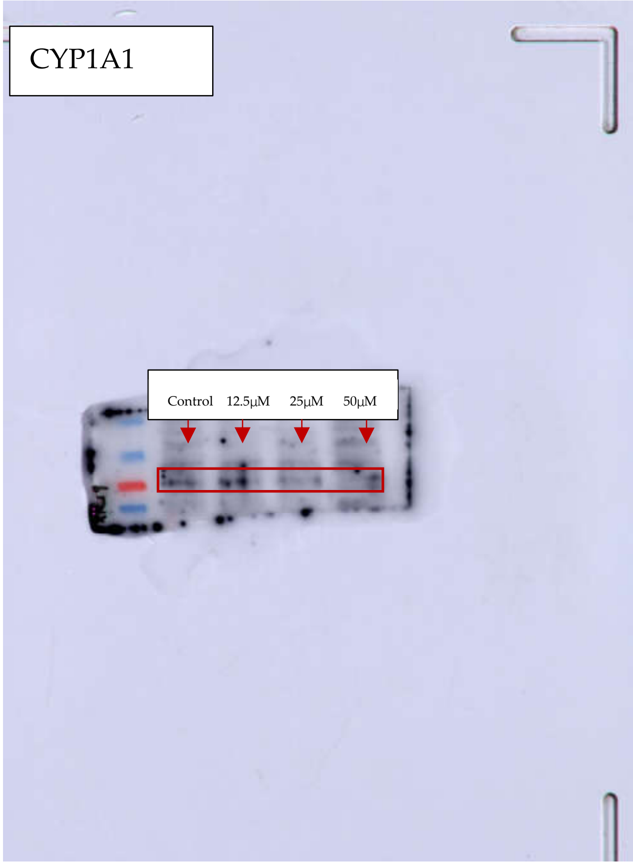
CYP1A1
Control
12.5μM
25μM
50μM

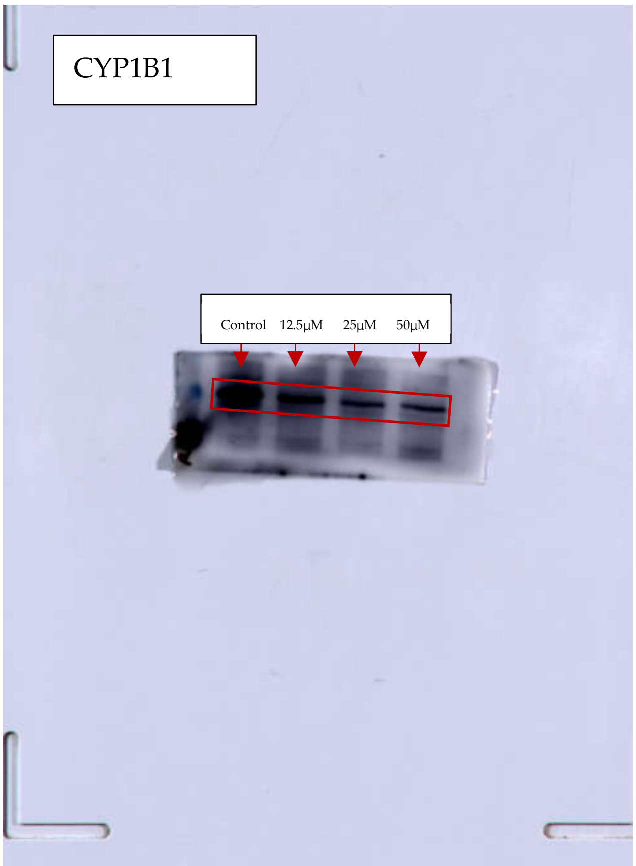
CYP1B1
Control
12.5μM
25μM
50μM
