# Supplementary figures and images for: Exosomes Derived from Yak Follicular Fluid Increase 2-Hydroxyestradiol Secretion by Activating Autophagy in Cumulus Cells
Source: Animals (Basel). 2022 Nov 16;12(22):3174. doi: 10.3390/ani12223174 (PMC9686841; doi:10.3390/ani12223174)

## Identification of yak follicular fluid exosomes

TSG101

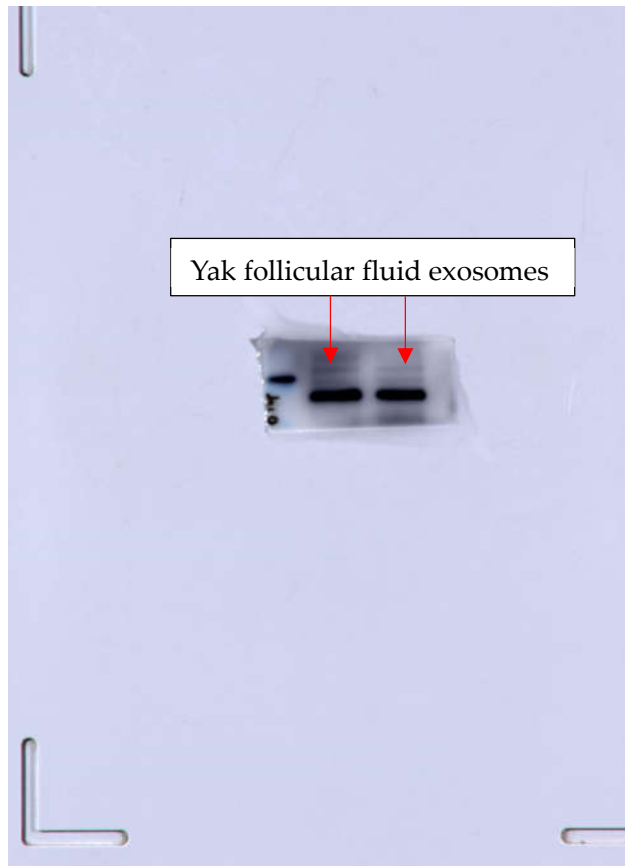

CD63

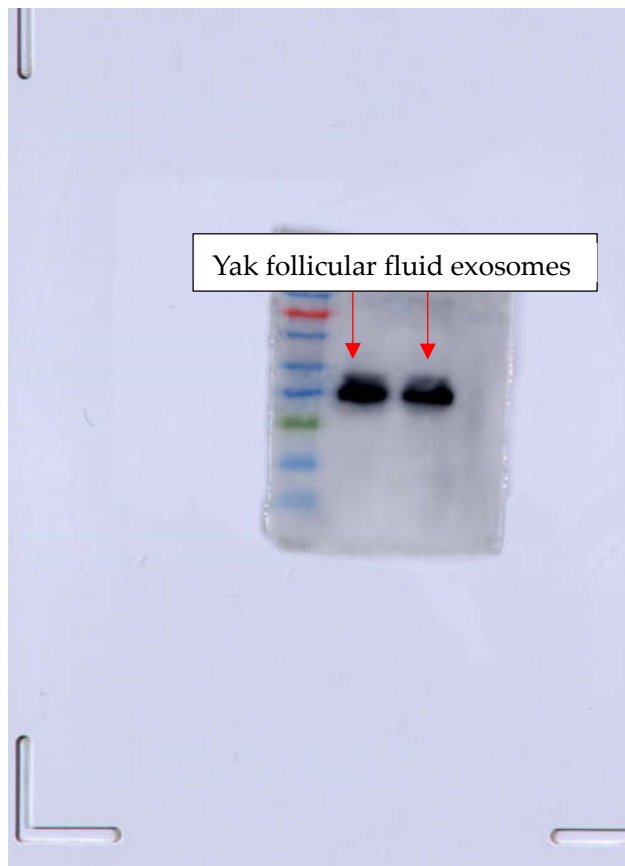

Supplement: Supplementary file 1 [file animals-12-03174-s001.zip › Figure S1 .pdf]
